# Bioactivity Enhancement of Plasma-Sprayed Hydroxyapatite Coatings through Non-Contact Corona Electrical Charging

**DOI:** 10.3390/nano13061058

**Published:** 2023-03-15

**Authors:** Pedro R. Prezas, Manuel J. Soares, João P. Borges, Jorge C. Silva, Filipe J. Oliveira, Manuel Pedro F. Graça

**Affiliations:** 1 I3N and Physics Department, University of Aveiro, 3810-193 Aveiro, Portugal; 2 I3N-CENIMAT, New University of Lisbon, 1099-085 Lisbon, Portugal; 3 CICECO and Materials Engineering Department, University of Aveiro, 3810-193 Aveiro, Portugal

**Keywords:** corona charging, non-contact electrical charging, hydroxyapatite coatings, atmospheric plasma spray, bioactivity

## Abstract

Atmospheric plasma spray (APS) remains the only certified industrial process to produce hydroxyapatite (Hap) coatings on orthopaedic and dental implants intended for commercialization. Despite the established clinical success of Hap-coated implants, such as hip and knee arthroplasties, a concern is being raised regarding the failure and revision rates in younger patients, which are increasing rapidly worldwide. The lifetime risk of replacement for patients in the 50–60 age interval is about 35%, which is significantly higher than 5% for patients aged 70 or older. Improved implants targeted at younger patients are a necessity that experts have been alerted to. One approach is to enhance their bioactivity. For this purpose, the method with the most outstanding biological results is the electrical polarization of Hap, which remarkably accelerates implant osteointegration. There is, however, the technical challenge of charging the coatings. Although this is straightforward on bulk samples with planar faces, it is not easy on coatings, and there are several problems regarding the application of electrodes. To the best of our knowledge, this study demonstrates, for the first time, the electrical charging of APS Hap coatings using a non-contact, electrode-free method: corona charging. Bioactivity enhancement is observed, establishing the promising potential of corona charging in orthopedics and dental implantology. It is found that the coatings can store charge at the surface and bulk levels up to high surface potentials (>1000 V). The biological in vitro results show higher Ca^2+^ and P^5+^ intakes in charged coatings compared to non-charged coatings. Moreover, a higher osteoblastic cellular proliferation is promoted in the charged coatings, indicating the promising potential of corona-charged coatings when applied in orthopedics and dental implantology.

## 1. Introduction

Atmospheric plasma spraying (APS) remains to be the referenced and certified industrial coating deposition process to produce bioactive hydroxyapatite (Hap) coatings for orthopedic and dental implants [1]. The international standards that regulate the chemical and mechanical properties of these coatings, such as ISO 13779, ASTM F1147, etc., were developed taking into account APS coatings. In their review publication, McCabe et al. compile the typical values that commercial APS Hap coatings present regarding the requirements stipulated by the international standards [2].

Total hip replacements (THR) and total knee replacements (TKR) are the most commonly performed arthroplasties worldwide. Although they have satisfactory clinical outcomes in older patients, the same is not true for younger patients, as, for example, a recent and relevant population-based study demonstrates [3]. L.E. Bayliss et al. identified 63,158 patients who had undergone a THR and 54,276 patients who had undergone a TKR, and followed up the data concerning these patients, for a maximum of up to 20 years, to find out the lifetime risk of requiring revision surgery. They found that the risk of revision surgery in patients who had THR or TKR over the age of 70 was about 5%, with no difference between sexes. However, for patients younger than 70 years who had the arthroplasty, the lifetime risk increased considerably; up to 35% for men in the 50–60 age interval (in this case there are significant differences between sexes, the risk being higher for men). Women, in the same age range, had an increased risk of 20%. The median time to revision surgery for patients who had the arthroplasty younger than the age of 60 was only 4.4 years [3]. This is indeed a significant problem because the number of younger patients requiring arthroplasties is increasing rapidly worldwide. For instance, based on the U.S. National Center for Health Statistics, the number of THR interventions increased from 138,700 in the year 2000 up to 310,800 in 2010. THR interventions increased by 92% among patients aged 75 or older, but interventions also increased by 205% in patients aged 45 to 54 [4,5]. Thus, the number of revision surgeries is expected to increase dramatically. Moreover, after a revision procedure, the risk of second revision increases [6]. Such patients, besides suffering from the very painful process they will have to overcome, might become a significant burden to society in terms of cost and disability [6].

Taking into account this problem, medical experts have been warning in the literature about the critical necessity for research and development in the field of implants which also takes into consideration the problem of younger patients as their expected lifetime is higher [7].

Among the reported methods to increase the bioactivity level of Hap, electrical polarization/charging is the one with the most outstanding results [8,9]. Biological studies have shown that electrically charged Hap is able to bond in vivo with the host bone in approximately half of the time compared to non-charged Hap, thus speeding up osseointegration and improving stability by stimulating the osteoblastic cellular activity and maximizing important parameters, such as the area of direct bone contact between the coating and host tissue [10,11]. Additionally, negative charges were demonstrated to produce a higher osseointegration enhancement compared to positive charge densities [12].

The electrical charging/polarization of Hap biomaterials has been essentially reported on bulk samples with parallel faces (pellets, etc.) because it is possible to charge them using conventional thermoelectrical poling methods [13]. However, this is not the case for the coatings. APS coatings have micrometric surface roughness values; thus, although it is simple to apply electrodes on the flat surfaces of the bulk Hap samples, it is a significant challenge on the coatings. Pressure, paint-on, or sputtered electrodes cannot be applied for many reasons. For example, if both sputtered or paint-on electrodes were not removed after electrical polarization/charging, they would obviously alter the expected bioactivity and cellular response in vivo. If they were removed by a polishing procedure, then the micrometric roughness of the coating, which is essential for the in vivo mechanical interlocking between the coating and host bone, i.e., for the implant mechanical stability, would be removed. Several other problems could be listed, such as the fact that the implants themselves do not have simple planar geometry.

This work attempts to demonstrate the increase in bioactivity in Hap coatings, for both orthopedics and dental implants, by using a non-evasive electrical charging technique: corona charging.

## 2. Materials and Methods

### 2.1. The Corona Triode

The corona discharge technique has been extensively used since the seventies, among other applications, in the cost-effective large-scale production of mainly polymer-based electrets. A concise description of our experimental corona triode setup, highlighting the most important features is presented in this section. A more detailed description, including photos of the experimental setup, can be found in our previous publication [14].

A corona discharge is a stable, self-sustaining partial electric discharge occurring in an insulating medium (e.g., air, argon, nitrogen, carbon dioxide, etc.), when a high dc potential, above a threshold value, is applied between asymmetric electrodes, typically, between a point and a plane. Potentials in the range of 10–15 kV are usually applied, and the polarity of the discharge can be positive or negative, depending on the polarity of the potential applied in the point electrode [15,16]. The discharge is perfectly controllable, thus it can be used as a source of low-energy thermalized ions to charge dielectric materials [16]. After multiple collisions in their path toward the material, the generated ions do not have enough energy to damage or penetrate the material; they just transfer their excess charge to the material’s surface [16].

**Figure 1:**
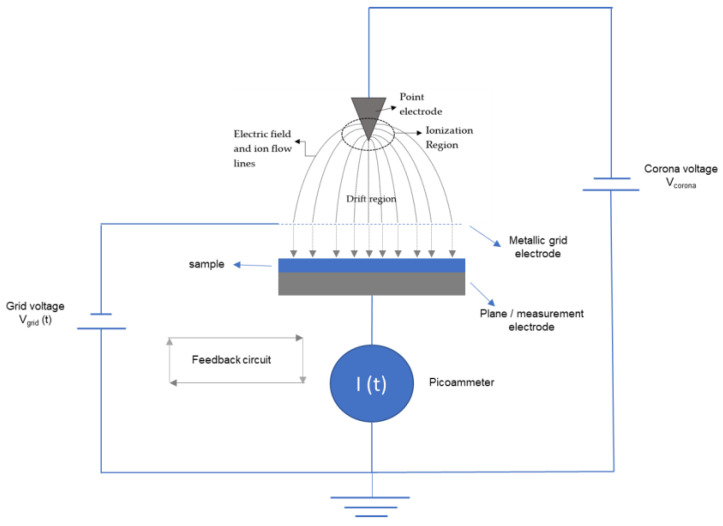
A corona triode employs three electrodes: the point, grid and plane/measurement electrodes, as indicated in the figure. In more advanced setups, the feedback circuit between the current I(t) flowing through the sample and the potential V_grid_(t) applied to the grid makes it possible to follow the sample surface potential buildup in real time during the charging process [14].

A corona triode scheme is shown in Figure 1. The triode designation refers to the presence of three electrodes: the point, the grid and the measuring electrode, as depicted in Figure 1. Two voltage sources are used: one controls the potential, V_corona_, applied to the point electrode, originating the corona discharge, and the other controls the potential V_grid_(t) in the grid electrode. In more advanced setups, the corona triode works in constant charging current mode, which makes it possible to determine and follow the sample surface potential buildup in real time while it is being charged. In this mode, V_corona_ is fixed during the experiment and the sample is charged with a constant current, usually within a few or dozens of nA range [17]. The constant current mode is implemented through the feedback circuit represented in Figure 1: while the sample is being charged, the V_grid_(t) value is being constantly updated so that the charging current flowing through the sample I(t), measured by the picoammeter, is approximately constant at the user-defined value I_0_. By doing this, it can be shown that the potential difference between the grid V_grid_(t) and the sample V_sample_(t) surface will have an approximately constant value ΔV_gap_ and that V_sample_(t) is given by [18,19]:

(1) $$V_{\mathrm{sample}}\left(t\right)=V_{\mathrm{grid}}\left(t\right)-\Delta V_{\mathrm{gap}}$$

The ΔV_gap_ term is determined without a sample on the measuring electrode for the defined constant current charging value I_0_ and other system parameters (temperature, distances between the point, grid and sample, polarity of the discharge, and humidity). The ΔV_gap_ determination is known in the literature as obtaining the calibration curves of the system. A detailed explanation can be found in Giacometti et al. [15].

The charging experiments were performed using a custom-made experimental setup. The charging temperature was set at 200 °C and the atmospheric relative humidity was kept lower than 10% in all the experiments. The V_corona_ potential was set at −15 kV in all the experiments and the distances between point electrode/sample and grid/sample were 70 mm and 4 mm, respectively. The samples were charged negatively with an approximately constant current of I_0_ = −1 nA.

### 2.2. Sample Preparation

The APS Hap coatings were provided by BioCeramed S.A. (Lisbon, Portugal), a European private company, which specializes in the production of Hap coatings for medical devices. The coatings were produced on square titanium grade 2 substrates with a side length of 25 mm and a thickness of 1 mm. The average thickness of the coatings is 70 µm. It is worth pointing out that commercial APS Hap coatings typically have thicknesses in the 50–100 µm range.

The Hap powder produced by BioCeramed S.A., and used in this study, is characterized by: Ca/P ratio is 1.667 ± 0.001, the crystalline phase composition (in wt%) is Hap 99.8 ± 0.2, β-TCP 0.2 ± 0.1 and CaO 0.1 ± 0.1. β-TCP and TTCP are not detected. The crystallinity ratio is 101.1 ± 2.2% and the particle size is distributed around 43.0 µm: d(0.1): 29.5 µm, d(0.5): 43.0 µm and d(0.9): 54.3 µm. No trace elements, such as Cd, As, Hg and Pb can be found. The specific plasma spray process parameters are intellectual property of BioCeramed S.A. and are therefore not available.

It is important to mention that the Hap powders used for this application can be produced by both top-down and bottom-up methods, namely, by the sol-gel method and by the solid-state reaction through high-energy mechanical milling processes, respectively. The co-precipitation method is another bottom-up process, which is the one used by Bioceramed S.A. because of the industrial advantages that it presents [20,21,22,23,24]. The Hap powder used meets the ISO 13779-1 and ISO 1379-2 standards.

### 2.3. Structural and Morphologic Characterization

The XRD results were obtained at room temperature on a Panalytical Empyrean diffractometer (Malvern, UK). CuKα radiation (λ = 1.54056 Å) was used and it was working at 40 kV and 45 mA. The scanning parameters were a scan step of 0.0260°, with a scan step time of 117 s, at room temperature and with a 2θ angle range between 10° and 74°.

The morphology was evaluated using an Vega-3 SEM microscope from TESCAN (Brno, Czech Republic). The sample’s surfaces were covered with carbon prior to the experiment to enhance the surface electron conductivity.

### 2.4. In Vitro Biological Tests

A total of 26 samples were used in the biological tests, 13 of which were negatively charged with a constant current of I_0_ = −1 nA and the other half was not charged (control samples). Of the 13 charged samples, 7 were used in the SBF tests and 6 were used in the osteoblast-sample interaction tests.

#### 2.4.1. Simulated Body Fluid (SBF) Immersion Tests

SBF tests were performed to study the variation, in function of the immersion time, of the Ca^2+^ and P^5+^ ionic concentrations, as well as the pH values. For the SBF preparation, 750 mL of high-purity deionized water was introduced into a 1 L capacity glass beaker. A magnetic stirrer was also introduced into the beaker and the system was placed on a hot plate/magnetic stirrer at 37 °C under a constant stir rate. Subsequently, the following reagents were added in the following order: 7.996 g of NaCl, 0.350 g of NaHCO_3_, 0.224 g of KCl, 0.228 g of K_2_HPO_4_·3H_2_O, 40 mL of 1 M HCl, 0.0278 g of CaCl_2_, 0.071 g of Na_2_SO_4_, 6.057 g of (CH_2_OH)_3_CNH_2_ and 0.305 g of MgCl_2_·6H_2_O. Afterward, the pH was measured and adjusted to 7.4, using 1 M HCl. Lastly, the volume of the solution was adjusted to 1 L and the pH was verified again to maintain the 7.4 value. Each sample was immersed in a glass container with SBF solution for a maximum period of 3 days. The ratio between the sample surface area and volume of SBF solution was defined to be 0.0075 cm^−1^. The glass containers remained inside an incubator at 37 °C. The concentration of the Ca^2+^ and P^5+^ ions and the pH values were obtained for different immersion times: 0, 1, 3, 6, 12, 24, 48, and 72 h. For each immersion time, 0.5 mL of solution was removed and ICP-AES (inductively coupled plasma atomic emission spectroscopy) measurements were performed.

#### 2.4.2. Osteoblasts—Sample Interaction Tests

Concerning the osteoblastic proliferation and metabolic biological tests, to analyse the cell-surface interaction, human osteoblasts (SAOS-2 cell line, ATCC, American Type Collection, ref. HTB-85) were seeded on the charged and non-charged surfaces. The cell culture medium consisted of McCoy’s 5A (Sigma-Aldrich, Burlington, MA, USA, #M4892) supplemented with 2.2 g/L sodium bicarbonate (Sigma-Aldrich, Burlington, MA, USA, #S5761), penicillin (100 μg/mL) and streptomycin (100 µg/mL) (Invitrogen, Waltham, MA, USA, #15140122) and 10% FBS (Fetal Bovine Serum, Invitrogen, Waltham, MA, USA, #10270106). Cultures were maintained in a CO_2_ incubator (Sanyo, Moriguchi, Japan, MCO10AICUV).

Samples were sterilized with ethanol 70% for 10 min and left to dry. Samples were then placed in a 12-well tissue culture plate (Sarstedt, Sarstedt, Germany), which was pre-wetted with a culture medium. Cells were seeded at a density of 3 × 10^4^ cells per cm^2^. Cell controls were set by seeding cells at the same density directly over the surface of the tissue culture plate (TCP) wells.

##### Osteoblastic Proliferation

The cell adhesion ratio was determined by evaluating the cell population 24 h after seeding and proliferation rates were determined by evaluating cell population every other day. Cell viability was assayed using a resazurin (Alfa Aesar, Haverhill, MA, USA) solution (0.04 mg/mL in PBS) as a cell viability indicator. Viable cells reduce resazurin (with an absorption peak at 600 nm) to resorufin (with an adsorption peak at 570 nm). For the assay, all media were replaced by a 1:1 mix of complete medium with the resazurin solution. This medium was also dispensed in wells without cells to be used as a reference. After 2 h 20 min of incubation in the CO_2_ incubator, medium absorbance was measured at 570 nm with a reference wavelength of 600 nm (Biotek, Winooski, VT, USA, ELX 800UV microplate reader). The corrected absorbance (obtained by subtracting the absorbance measured at 600 nm from the one measured at 570 nm and subtracting the medium control) is proportional to cell viability. The combined standard uncertainty was calculated by the propagation of uncertainties.

##### Morphology and Vinculin Expression

To observe cell morphology, cells were stained after 5 days in culture. Cells were fixed with 3.7% paraformaldehyde, permeabilized with Triton X-100 (0.5% in PBS) and blocked (to avoid non-specific staining by the secondary antibody) with a 1% bovine serum albumin solution containing 0.2% Triton X-100. Cells were then immunostained with the primary antibody against vinculin, a focal adhesion protein (Anti-Vinculin antibody, Mouse monoclonal, clone hVIN-1, purified from hybridoma cell culture, Product Number V9264, Sigma-Aldrich, Burlington, MA, USA) followed by the secondary antibody (Goat anti-Mouse IgG (H+L) Cross-Adsorbed Secondary Antibody, Alexa Fluor 488, Catalog #:A-11001, Molecular 142 Probes, Thermo Fisher Scientific, Waltham, MA, USA). To observe the F-actin cytoskeleton, cells were stained with Acti-stain 555 Phalloidin (100 nM in PBS) (Cat. # PHDH1, Cytoskeleton, Inc., Denver, CO, USA) and to observe nuclei, cells were stained with DAPI (4′,6-Diamidino-2-Phenylindole, Dilactate, Cat # D3571, Invitrogen, Waltham, MA, USA: 300 nM in PBS). All samples were mounted on glass coverslips with fresh PBS and imaged with an epi-fluorescence microscope Nikon Ti-S. The microscopy of the tick film cross-section and the Hap film surface with 0 h of immersion (control) was performed in a TESCAN-Vega3 scanning electron microscope.

### 2.5. Photoelectron Emission Spectroscopy

Photoelectron emission spectroscopy measurements were performed to determine the electron work function necessary to eject an electron from the sample surface. Measurements were performed using a custom-made spectrometer [25].

Each sample was measured separately at two different spots on the sample surface. Three measurements were taken per spot. Measurements were performed after reaching a vacuum of 10^−5^ Pa inside the vacuum chamber. Photostimulation was performed using a 30 W deuterium light source. For incident light wavelength selection an MSD-2 monochromator was used. For the photoelectron current detection, a SEM-6 secondary electron multiplier with a grid voltage of 600 V and a multiplier tube voltage of 3.5 kV combined with a Robotron 20 046 pulse counter and a Hamamatsu Pulse Counting Board were used. The photoelectron current was scanned in a 3–6 eV range of photostimulation.

## 3. Results and Discussion

### 3.1. Structural and Morphological Analysis

**Figure 2:**
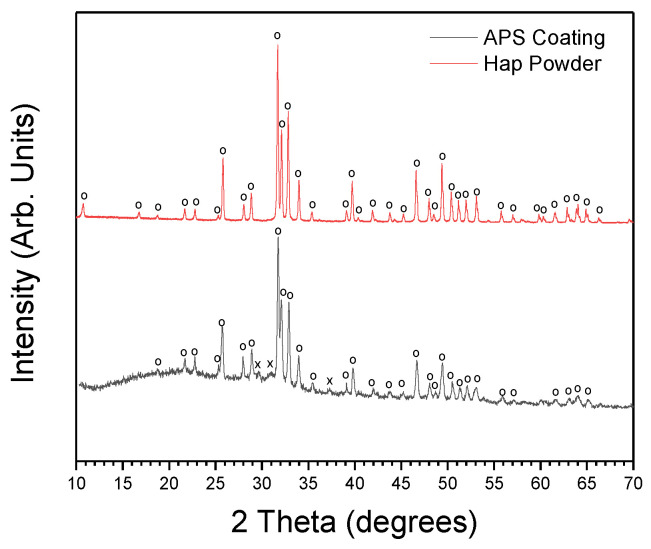
XRD diffractogram of Hap powder used, from Bioceramed S.A (Hap (ICDD ref.: 01-084-1998)) and of APS Hap coating. Hap and β-TCP crystalline phases are detected, including the habitual amorphous halo observed in APS coatings (O-Hap (ICDD ref.: 01-084-1998); X-β-TCP (ICDD ref.: 04-006-9376)).

XRD measurements were performed to identify the crystalline phases present within the APS Hap coatings. These measurements were performed using a PANalytical Empyrean Powder X-ray diffractometer. Figure 2 displays the X-ray diffractogram of the Hap powder used in the APS process and the diffractograms of the coating. The results are in agreement with the literature [26]. Hap and β-tricalcium phosphate (β-TCP) crystalline phases are detected, including the typical amorphous halo, which can be ascribed to amorphous calcium phosphate (ACP). Sometimes the designation “amorphous hydroxyapatite” can be found in the literature and is associated with the amorphous halo, but the correct designation is amorphous calcium phosphate. The very high temperatures experienced by powder particles during APS deposition promote their partial melting and the subsequent high cooling rates lead to the presence of a glassy phase within the coatings [1,27].

**Figure 3:**
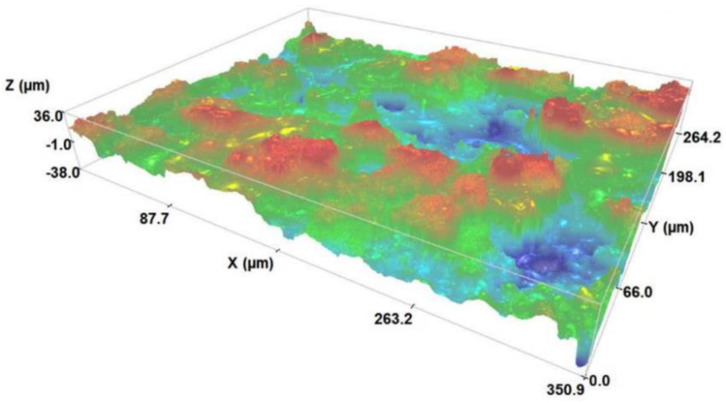
A 3D topographical surface map of an APS Hap coating showing the rough surface of the samples.

Additionally, optical profilometry measurements were also performed to determine the average surface roughness (Sa) of the coatings. These measurements were carried out using a Sensofar S Neox 3D optical profiler. It was found that the Sa values are 10 ± 1 µm mark. A 3D surface topographical map of a coating is presented in Figure 3 showing the rough surface of the samples. It should be noted that this map is very representative of the results obtained in different sample surface zones and in similar samples produced under the same conditions, which confirms the advantage of this method in the thickness and roughness reproducibility of the deposited film. This measurement was performed in 10 APS Hap-coated samples.

### 3.2. Corona Triode Charging Experiments

In our previous work, corona charging experiments were made on Hap bulk samples (pellets) [14]. In those samples, due to their planar faces, after the charging experiments, electrodes can be applied and carried out in thermally stimulated depolarization current (TSDC) measurements in order to determine the stored charge density and estimate its temporal stability [28]. The results showed very promising stabilities, with more than one year required for the stored charge to discharge [14]. Additionally, the stored charge densities were also very high, in the 10^−4^ C/cm^2^ magnitude, one or two orders of magnitude higher than the typical values reported for the conventional thermoelectrical polarization of Hap bulk samples [12].

**Figure 4:**
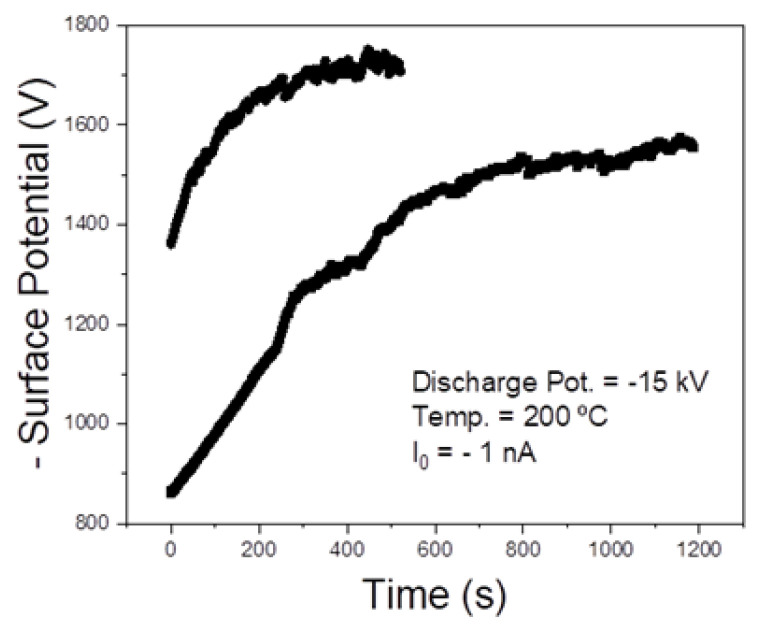
Example of two surface potential buildup curves for two APS Hap coatings negatively charged with a constant current of −1 nA.

This work makes the transposition from bulk samples [14] to APS coatings. In this case, TSDC measurements cannot be made because of the high surface roughness values (Figure 3). However, as presented later in this article, in vitro biological tests were carried out, which are a good indirect way of accessing the effects of the stored electric charge and comparing charged and non-charged samples. Furthermore, one of the advantages of the constant charging current method is that, despite not providing information about the temporal stability of the stored charge, it makes it possible to know if the material is effectively being charged. Figure 4 presents two surface potential buildup curves for two APS Hap coatings charged with a constant current of I_0_ = −1 nA. The shape of the curve is the same for all the samples: an initial approximately linear behaviour and subsequently a sublinear behaviour where the potential raises at a slower rate up to the saturation value, where it stabilizes. Such a curve shape has been generally observed in materials treated by corona charging, such as Teflon polymer foils [29]. Concisely, the physical meaning of the curve shape is related to the current flowing through the sample [29]. When the surface potential increases approximately linearly, it means that the electrons supplied by the corona discharge are trapped at a surface level in the sample. In the sublinear region, before saturation, the surface potential buildup rate decreases because now electrons are being injected into the sample, meaning that the charges can be trapped in both the surface and bulk zones. Finally, in the saturation region, charge trapping might become negligible because the current in the sample becomes purely conductive, without a displacement current density contribution. Another possible option in the saturation region is that the electrons are still being trapped in the bulk activating an intrinsic polarization mechanism of the sample [19]. Prezas et al. [14] demonstrated that this last option occurs in the Hap bulk samples. Ferreira et. al. in [19] explain, in a detailed form, the theoretical models behind the interpretation of surface potential buildup curves under the constant charging current regime.

Another aspect is that, as would be expected, different samples may reach different surface saturation potentials. The saturation potential is related to the amount of charge that can be stored at a surface level. Given the “violent” nature of the APS process, although macroscopic properties of the coatings can be considered reproducible (thickness, average surface roughness, adhesion strength, etc.), more specific properties such as surface area and trap density may not be reproducible, which explains the variance. Nonetheless, the saturation potential of almost all of the charged coatings fell within the −1400–1800 V interval. An interesting area for future research would be to perform charging experiments at room temperature (RT) to see if similar results can be obtained, which is also a known advantage of corona charging. For instance, for a ferroelectric polymer such as β-PVDF, although it was originally thought that temperatures up to 150 °C were required for a successful polarization, the first applications of the corona triode demonstrated that a large and stable polarization can be achieved at RT [30]. It must be highlighted that conventional polarization of Hap is usually carried out at temperatures in the 350–500 °C range, with applied electric fields in the 1–5 kV/cm range [31,32].

Before addressing the in vitro biological test results, an explanation about the reason why the samples were charged with a current value of I_0_ = −1 nA must be given. As previously mentioned, typical corona charging currents are within the few or dozens of nA range [19]. From the work presented in [14], it is known that lower charging current values tend to yield higher surface potential saturation values. This is related to the fact that higher charging currents create instability in the coatings and localized discharges in the bulk of the samples, which, as it was observed in the experiments in [14], prevents higher surface potentials from being reached. Such localized discharges have also been reported in the literature [33]. In a previous work [14], it was observed through photoelectron emission spectroscopy that bulk samples charged with lower constant current values have a higher electronic work function, i.e., more energy is required to eject an electron from the surface, which implies that they have a higher surface potential, which is in agreement with the corona charging experiments. In the case of the coatings, the same behavior was observed, as can be attested in Table 1: lower charging current values are associated with higher electronic work functions. It must be noticed that photoelectron spectroscopy is a surface characterization technique because UV radiation does not penetrate more than a few nanometers of the material [34].

### 3.3. In Vitro Biological Results

As mentioned in Section 2.4, a total of 26 samples were used on these measurements, 13 of which were negatively charged, and the other half were not charged (control samples). It is relevant to mention the following fact: the time gap between the charging of the coatings and the start of the biological tests was one month. According to the TSDC results presented in a previous report regarding bulk samples (pellets) [14], one month is not expected to be significant in terms of the discharge of the stored charge in the samples. Thus, this is a good opportunity to demonstrate the stability of the stored charge in the coatings.

#### 3.3.1. SBF Immersion Tests

**Figure 5:**
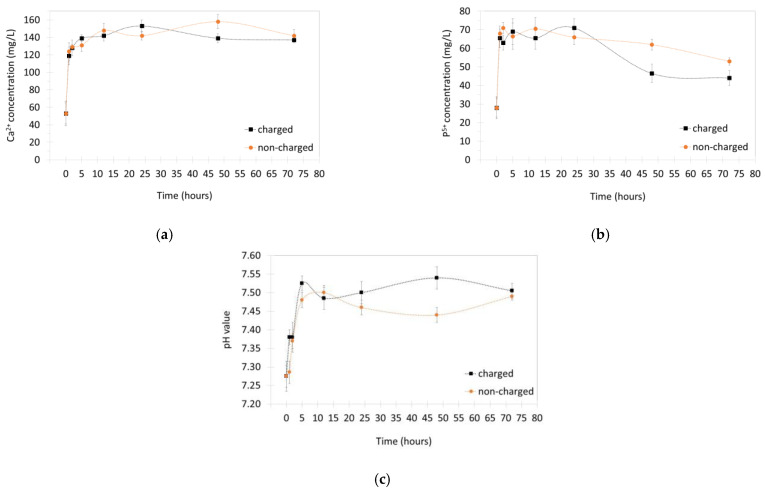
The variation of the Ca^2+^ (**a**) and P^5+^ (**b**) ionic concentrations and of the pH value (**c**) for increasing immersion times in the SBF solution (0, 1, 3, 6, 12, 24, 48 and 72 h), for the charged and non-charged samples. The first value at 0 h is equal for both samples, matching the initial concentrations and pH value of the SBF solution.

Figure 5 displays the variation of the Ca^2+^ and P^5+^ ionic concentrations for increasing immersion times in the SBF solution (0, 1, 3, 6, 12, 24, 48, and 72 h), for the charged and non-charged samples. The results follow the same trend reported in the literature for APS Hap coatings [35], i.e., an initial strong increase of the Ca^2+^ and P^5+^ ionic concentrations, and subsequently, their concentration starts to decrease. The initial increase in concentration is caused by the partial dissolution of the Hap coating. It is well known that the APS process leads to a reduction in the Hap crystallinity within the coating, as well as to the presence of secondary phases such as β-TCP and ACP. These secondary phases, with lower Ca/P ratios compared to Hap, have lower stability under the physiological conditions and therefore, they dissolve faster. This is the reason why calcium phosphate compounds with Ca/P ratios lower than 1.5 are not applied in orthopaedics; their degradation kinetics are faster than the rate of new host bone formation [36]. Regarding the crystallinity factor, it is also well known from the literature that the dissolution rate in physiological conditions increases with the decrease of the crystallinity degree of the material [37,38].

After the initial strong ionic concentration increase, one hour after immersion, the concentrations start a stabilization trend behaviour. For the higher immersion times, 48 and 72 h, there is a significant decrease in the Ca^2+^ and P^5+^ ionic concentrations, suggesting that the development of a carbonated bone-like Hap layer is taking place at a fast rate. It should be noted that, according to the literature, the development of the bone-like layer is most likely taking place for shorter immersion times, already a few hours after immersion [39]. However, a decrease in the Ca^2+^ and P^5+^ concentrations is not visible because it is “masked” by the partial dissolution of the coating. The stabilization trend of the concentrations a few hours after immersion is due to the fact that the coating surface is starting to be completely covered with a developing bone-like Hap layer, thus, completely blocking the dissolution.

**Figure 6:**
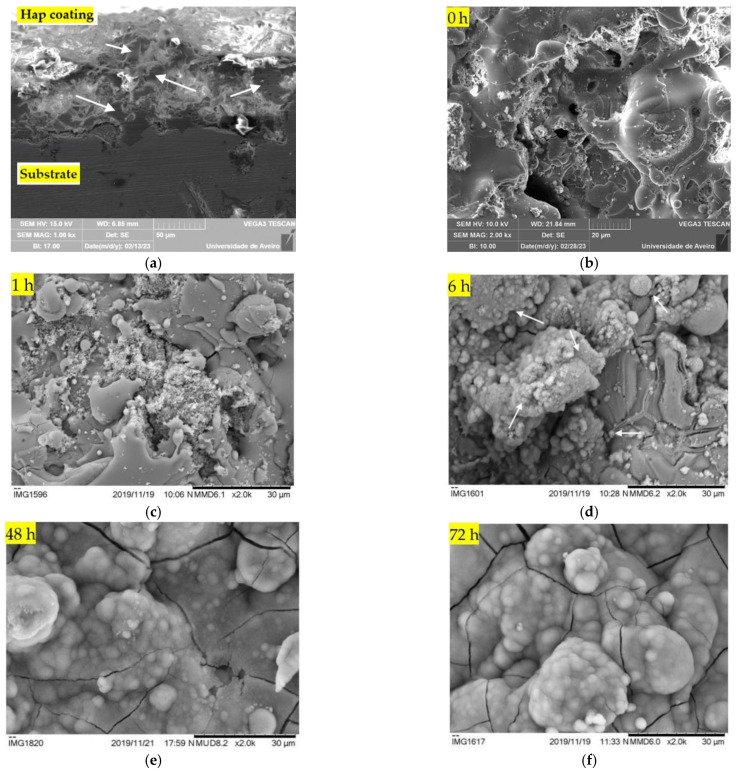
SEM micrographs revealing the cross-section of the Hap coating (**a**) and the surface morphology of the charged coatings for different immersion times: 0—no immersion (**b**), 1 (**c**), 6 (**d**), 48 (**e**) and 72 (**f**) hours. The arrows reveal bone-like carbonated Hap structures formation.

The SEM micrographs displayed in Figure 6 show the surface morphology of the charged coatings for different immersion times: 0 (6b), 1 (6c), 6 (6d), 48 (6e), and 72 h (6f). After 1 h, it is observable that there are already structures precipitating on the surface of the coating. At 6 h, the typical cauliflower-like structures assigned to the precipitation and development of a bone-like carbonated Hap layer are visible (the arrows point to some of these structures). These structures do not uniformly cover all the coating surface, being more developed in some regions and less pronounced in other regions. This behaviour is also typically observed in the literature [40]. At 48 and 72 h, a radical modification of the initial surface morphology is evident, with the top surface being completely covered with the newly forming Hap. These results show that the formation of new Hap is taking place at a fast rate thus proving the high bioactivity level of the coatings. A correlation between these results and the variation of the ionic concentration, presented in Figure 5, can be seen. In the previous paragraph, it was mentioned that the Ca^2+^ and P^5+^ concentration decrease for lower immersion times is “masked” by the partial dissolution of the coating, but for higher immersion times a decrease is visible, suggesting that all the coating surface is covered by new Hap. This line of thought is corroborated by the micrographs in Figure 6, which show an incomplete surface coverage for lower immersion times and a complete coverage for 48 and 72 h. Figure 6a also shows a micrograph of the cross-section of a Hap coating. As expected, the thickness of the layer is around 70 µm. A high surface rugosity is also visible. In this picture, the arrows indicate the possible partial melting zones, as detected in the XRD diffractogram by the presence of an amorphous halo.

The mechanism of new carbonated Hap formation in SBF, including the importance of the electrostatic interactions, is explained in Kim et al. [39]. Accordingly, if the electrostatic interactions are assumed to be important even for non-charged Hap, the influence of an additional large stored negative charge becomes clearer: it should accelerate the process. Thus, as Figure 5 demonstrates, for the higher immersion times, the Ca^2+^ and P^5+^ concentration decrease is higher on the charged coatings, indicating a more advanced stage of bone-like Hap development.

Finally, the variation of the pH displayed in Figure 5 shows an initial increasing trend followed by a stabilization behaviour. This behaviour is also typically observed in the literature for Hap-based materials that present some degree of dissolution in the SBF solution [38,40,41,42,43]. Such behaviour is commonly explained by the release of alkaline earth Ca^2+^ ions and also OH-ions from the Hap coating which increase the pH value. Furthermore, some ionic exchange between ions released by the coating with ions present in the SBF solution can contribute to the pH increase [38,42,43].

#### 3.3.2. Osteoblasts—Sample Interaction Tests

##### Osteoblastic Proliferation and Vinculin Expression

**Figure 7:**
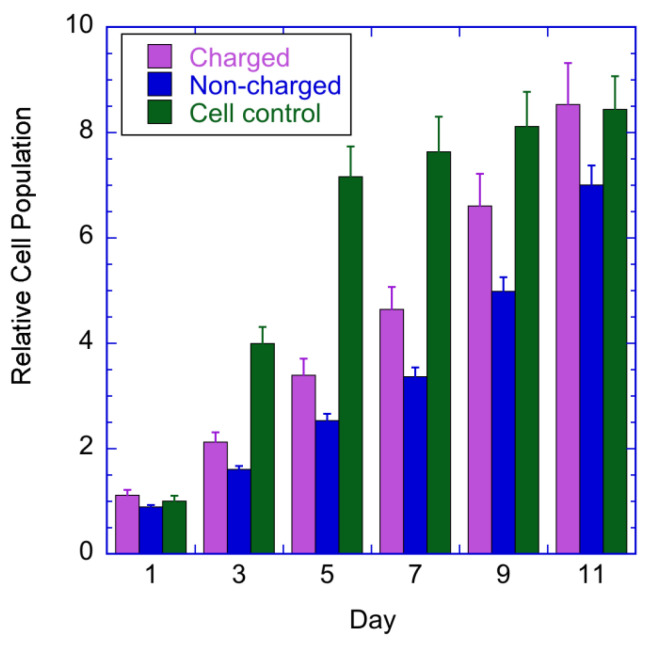
Osteoblastic cellular proliferation in the charged and non-charged coatings for increasing culture times.

The results contained in Figure 7 display the osteoblastic cellular proliferation in the charged and non-charged coatings for increasing culture times. Firstly, the control population exhibits an expected behaviour: a fast-initial growth and later, a slower rate due to the stabilization of the number of cells in the area of the culture plate well. It is also normal for the control population to increase faster than the samples in the first days. The sample results indicate that, for all the culture times, the osteoblastic proliferation is higher in the charged coatings, compared to the non-charged coatings, and becomes more evident for higher culture times (also observed in the literature, for instance, Bodhak et al. [12]). Therefore, despite the fact that the biological tests started one month after the corona charging experiments, the stored charge is clearly able to stimulate osteoblastic proliferation.

**Figure 8:**
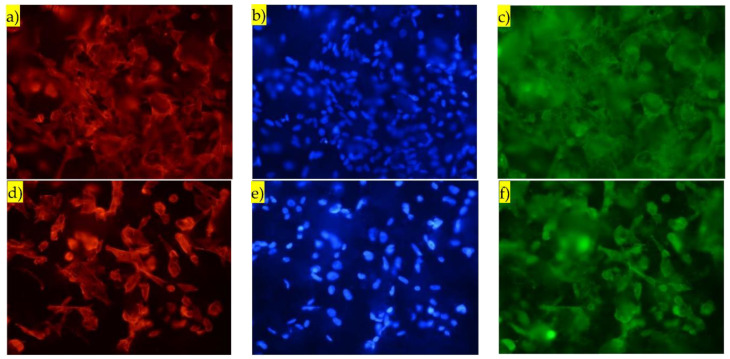
Immunofluorescence images obtained five days after culture. The red fluorescence indicates the cytoskeleton of the osteoblasts, the blue fluorescence indicates the osteoblasts’ nuclei and the green fluorescence indicates vinculin protein. Images (**a**–**c**)—charged APS Hap coating; (**d**–**f**)—non-charged APS Hap coating. The images were obtained with the 40x objective, and correspond to an area of about 330 µm × 424 µm.

Figure 8 presents immunofluorescence images obtained by fixing and staining the osteoblasts after 5 days of culture in the coatings. The red fluorescence displays the cytoskeleton of the osteoblasts, the blue fluorescence displays the nuclei, and the green fluorescence displays the vinculin protein distribution. Images (a), (b) and (c)—charged coating; (d), (e) and (f)—non-charged coating. The images correspond to an area of about 330 µm × 424 µm. The immunofluorescence images in Figure 8 complement the resazurin proliferation information contained in Figure 7 by showing a clear and abundant osteoblastic proliferation in the charged and non-charged surfaces after 5 days of culture. The elongated morphology of the nuclei (blue fluorescence in Figure 8) shows that the osteoblasts adhere and interact positively with the coating surface. In surfaces where the osteoblasts do not adhere well, they instead take a round shape. The well-developed, projected and elongated cytoskeleton displayed in images (a) and (d) corroborate strong osteoblastic adhesion and positive interaction with the surface. If the adhesion and interaction were poor, a well-developed, projected cytoskeleton would certainly not be observable after 5 days of culture. Lastly, the well-distributed green fluorescence of the vinculin protein shows strong bonding and adhesion between the osteoblasts and the surface of the coatings. As a remark, the fluorescence microscope used in this work is not confocal, which explains why some out-of-focus spots are visible in Figure 8; the large surface roughness of the APS Hap coatings should also be taken into account.

It should be noted that a qualitative analysis of the fluorescence images in Figure 8 should be avoided, i.e., to compare images (a) and (d) and conclude that the osteoblastic adhesion is better in (a) compared to (d), or to compare (c) with (f) and conclude that the vinculin distribution is better in (c), i.e., the charged coatings. The conditions to be able to perform a qualitative analysis such as, for instance, the one contained in [12] are very hard to obtain; only in very strict conditions can such analysis be correctly and reliably performed. Specifically, it must be confirmed that the regions in Figure 8 contain the same cell density, which is very difficult considering the micrometric surface roughness of our coatings and also the fact that, in this case, a non-confocal fluorescence microscope was used. Therefore, the data contained in Figure 8 acts as complementary information concerning the proliferation results in Figure 7. Although Figure 7 shows an enhanced osteoblastic proliferation in the charged coatings, Figure 8 provides, after 5 days of culture, a visual confirmation of the osteoblastic proliferation and additionally, it confirms that the osteoblasts have a positive interaction with and strong adhesion to the surface of the coatings.

## 4. Conclusions

In this work, a novel concept of electrically charging APS Hap coatings on titanium substrates using a corona triode was introduced, making it possible to obtain a stable negative charge density on the coatings without mechanical contact. These coatings present an average surface roughness of about 10 µm. In vitro biological tests, started one month after corona charging, demonstrate that the stored charge promotes a higher osteoblastic proliferation compared to non-charged coatings as well as higher Ca^2+^ and P^5+^ ionic intake in SBF solution, indicating a faster bone-like Hap development on the charged coatings.

Corona charging makes it possible to charge the APS Hap coatings up to very high surface potential values that are easily higher than 1000 V for a discharge potential of −15 kV and charging current of −1 nA. Therefore, much higher electric fields across the samples than those achievable by conventional thermoelectrical polarization are obtainable, which is a well-known advantage of the corona charging of other materials such as polymer foils. Furthermore, lower charging current values tend to yield higher surface potential values and thus, higher stored charge densities at the surface level.

The osteoblastic cellular proliferation results for the charged and non-charged coatings show that charged samples present a cell population value >25% higher than the non-charged ones.

Additionally, the immunofluorescence images complement the osteoblastic proliferation results and show a well-developed osteoblastic cytoskeleton and a well-distributed vinculin signal, which are clear indicators of positive interaction and strong adhesion between the osteoblasts and the coatings.

One month after being corona charged, the biological tests show how the stable, negative charge density can enhance the bioactivity of the corona-charged coatings. These charged coatings with enhanced bioactive properties are in compliance with the international standards regulating the market and would offer a new, differentiated and advantageous solution that could provide better service and care for a continuously increasing number of patients.

## Figures and Tables

**Table 1:** The electron work function of APS Hap coatings charged with constant current values of −1 and −3.5 nA.

	−1 nA	−3.5 nA
Work function (eV)	4.83	4.76
Standard deviation (eV)	0.04	0.04

## Data Availability

Not applicable.
